# Enterobiasis in Ectopic Locations Mimicking Tumor-Like Lesions

**DOI:** 10.1155/2009/642481

**Published:** 2009-06-14

**Authors:** Silvio Pampiglione, Francesco Rivasi

**Affiliations:** ^1^Department of Veterinary Public Health and Animal Pathology, University of Bologna, 40064 Ozzano Emilia, Italy; ^2^Department of Pathologic Anatomy and Forensic Medicine, Section of Pathological Anatomy, University of Modena and Reggio Emilia, via del Pozzo no 71, 41100 Modena, Italy

## Abstract

Both the clinical and the histopathological diagnostic difficulties of oxyuriasis in unusual sites and their importance from a clinical point of view are pointed out. The authors report two ectoptic cases of enterobiasis observed in Northern Italy, one located in a fallopian tube of a 57-year-old woman and the other in a perianal subcutaneous tissue of a 59-year-old man, mimicking tumor-like lesions. The authors take advantage of the occasion to focus the attention of the medical world on this subject, lamenting the scarce importance given to this parasitosis in university courses of medical schools and in medical textbooks as it is incorrectly considered “out-of-fashion.”

## 1. Introduction

*Enterobius vermicularis * (Nematoda, Oxyuridae) is a very common parasite, perhaps the most common among the helminths which are considered strictly specific for man [1], even if it has been argued recently that *E. vermicularis *causes infection in captive chimpanzees [2–4]. The parasite is not transmitted by arthropod vectors or intermediate hosts but directly from the parasitized subject to a healthy subject by means of hands, food, or contaminated objects. An indirect transmission by air has also been shown for human enterobiasis, since its microscopic eggs are capable of being released in the air and inhaled with dust [5].

Its existence, well known since ancient times, is not clearly influenced by climatic differences so that the helminths can really be considered cosmopolitan, found from tropical zones [6] to polar zones [7] and from different socioeconomic situations [8]. In fact, it is present in the same region both in the rich and the poor. Even though it is prevalent in children, in particular in scholastic communities, it is, however, reported in subjects of all ages, sometimes mild and of scarce importance but other times with notable problems due to the frequent involvement of the coenesthesia, complications due to microbic infections as a result of lesions from scratching or not easily controlled chronic forms which can sometimes be complicated by psychoneurotic involvement. During the cycle of the parasite the female, once fertilized, descends into the rectum from its habitual location, the caecum and the colon, and lays its eggs in the anal folds and the surrounding areas. The deposited eggs mature in a few hours and contain fully developed larvae becoming infective. The movements of the females and the laying of the eggs cause itching, predisposing the passage of the infection to the same patient or other patients through hands which have been contaminated in this way. Parasitosis in these locations is easily able to be diagnosed by means of specific parasitological methods (perianal swabs or the Scotch cellophane tape test). Self-infestation is frequent and can cause long-term infections. It occurs either through one of the above-mentioned mechanisms or through so-called retrofection [9] and, therefore, with the hatching of the eggs outside the anal sphincter followed by a retrograde ascent into the rectum on the part of the emerged larvae [10]. Retrograde infection of *E. vermicularis* may occur only in particular condition, including perianal manipulation and anal sex, but is not the usual route. In female subjects, *E. vermicularis* can sometimes take another path and be found in the genitals, going from the vulva back up to the Fallopian tubes [11, 12], sometimes reaching the ovaries [13, 14] and also having the capacity of penetrating the surrounding peritoneum [15]. Other times, both in female and male patients, the parasite can go astray into rare ectopic locations such as the tissues of the perianal region [16–18], the prostate [19], the urinary bladder [20], the ureter [19], the spleen [21], the peritoneum [22, 23], the mucosa and/or the appendicular lumen [23, 24], the intestinal wall [25], the liver [26, 27], the lungs [28, 29], the epididymis [30], and the conjuntival sac [31]. In such cases, clinical diagnosis is impossible, and the diagnosis can only be reached histologically, generally representing an unexpected surprise for the histopathologist. With the report of two recent ectopic cases, one located in a Fallopian tube and the other in a perianal abscess, which recently came to our attention. we would like to point out these difficulties in the histopathological diagnosis of oxyurasis in nonhabitual sites, and their importance from a clinical point of view.

## 2. Cases Report

### 2.1. Case 1

A 57-year-old nomadic Yugoslavian woman without a fixed abode, was referred urgently to Voghera Hospital (Northern Italy) in January 2003 for an acute abdomen. From the anamnesis, collected with difficulty due to the language problems, it turned out that the patient had been suffering from painful crises in the lower abdominal quadrants for many years. As the pain had become unbearable, the patient asked for medical aid. When the abdomen was opened, the surgeon found a bilateral tubal-ovarian abscess, associated with diffuse peritonitis. Both adnexa were removed. In the left tubal wall, a nodular lesion was also discovered, hard in consistency, measuring 1 × 2 cm, under the peritoneal serous membrane.

**Figure 1:**
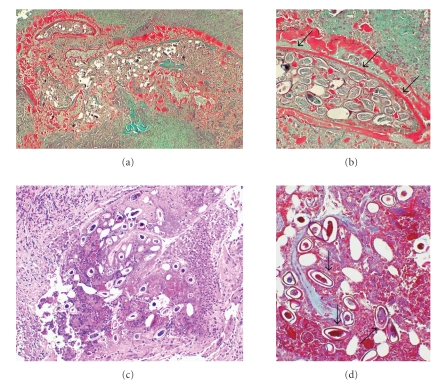
Histological aspects of ectopic locations of *E. vermicularis*: low-power view: (a) and (c) (cases 1 and 2, respectively) (Masson Goldner trichrome, original magnification x50 (a) and H&E x125 (c)). At higher magnification: (b) (case 1), numerous eggs oval in shape with a thick shell, sometimes calcified are seen, fragments remnants of the uterine wall of the nematode are still present (arrows) (Masson Goldner trichrome, original magnification x250). At higher magnification: (d) (case 2) in the center of suppurative tissue eggs-shaped formations at time.

At histological examination of the nodule, lymphocytic and plasmacellular chronic infiltration and a conspicuous number of eosinophils were observed at the periphery while amorphous material was present in the centre containing numerous eggs, oval in shape with one flattened side, sometimes calcified or altered by regressive process, measuring approximately an average of 50 *μ*m in length and 20 *μ*m in width, referable to eggs of *E. vermicularis* (Figure 1(a)). Other more or less linear structures, probable remnants in an advanced regression phase of the uterine wall of the nematode, were also visible at the periphery of the egg cluster (Figure 1(b)).

### 2.2. Case 2

A 59-year-old man, living in the country near Modena, was admitted to Vignola Hospital (Modena, Northern Italy) in March 2006 for perianal swelling which had been present for about a year but which had become highly painful about three weeks before, in particular while defecating. Fifteen years previously, the patient had undergone chemotherapy because he had a non-Hodgkin's lymphoma which had regressed but recurred after 8 years and was currently inactive under pharmacological control. On physical examination, a rounded mass having a diameter of about 2 cm, externally palpable in the posterior quadrants of the perineum, highly painful at palpation, having a hard-elastic consistency, with the skin moderately reddened was noted. Rectal exploration, which was painful, demonstrated that the mass was located in the perianal subcutaneous tissue. Neither fistulas nor rhagades were observed. The nodular tumor-like lesion, enucleated surgically through the skin was examined histologically and was composed of cutaneous and subcutaneous tissues with partially obliterated fistulous pathways which communicated deeply into the suppurative area with inflammatory and chronic infiltrations and with the presence of a plurinucleate giant cell granuloma, foreign body type, fibrous tissue, lymphocytes, and numerous eosinophilic granulocytes mingled with necrotic material (Figure 1(c)). In the centre of the suppurative tissue, egg-shaped formations, at times asymmetric, 52–55 × 20–25 *μ* in size, are found, which contain fragments of an amorphous substance, residue of elements altered by regressive process, sometimes calcified, and identifiable by the dimensions and form as *E. vermicularis* eggs (Figure 1(d)). Some elongated formations having a length of about 10–15 *μ* without cellular structure and present in the middle of the inflammatory tissue, stained blue by using the trichromic technique of Masson Goldner, may be remnants of the worm's wall, highly altered by the inflammatory process.

In the context of the fistula, minute pilar fragments and keratin lamellae remnants of a pilonidal cyst were found. Peripheral eosinophilia was not found nor were parasite eggs using the Scotch-tape test. Therefore, a diagnosis of pilonidal cysts with oxyuris eggs was made in an individual under chemotherapeutic treatment for non-Hodgkin's lymphoma.

## 3. Discussion

In the first case, it would seem, therefore, that the worm had gone back up the genitals and, after penetrating the thickness of the tubal wall which had probably already been damaged by other infective agents, it was halted by the defensive reaction of the host tissues and remained among the inflammatory cells. Similar cases have been reported over the last decade by many authors [15, 32–34]. Regarding the second case, the presence of *E. vermicularis* eggs in the context of the suppurative swelling makes it possible to hypothesize that a gravid female parasite re-ascended into the depth of the perianal tissue by means of a fistulous pathway and remained trapped by the occlusion of the pathway itself, dying and causing a granulomatous inflammatory reaction which caused the destruction of its structure, thus releasing the eggs in the phlogistic tissue. The state of evident immunodepression may most likely have favored this unusual location. From a review of the literature, location in the female genital tracts, considering as such also those peritoneals at the pelvic level (according to Symmers, 1950) [27] described from the end of the 1800s [9] to today are not, in reality, so rare as one might think; in fact, we were able to find more than 80 cases, reported in 24 different countries of 3 continents (France, UK, USA., Canada, Cuba, Italy, Spain, Germany, Czech Republic, Denmark, Sweden, Hungary, Tunisia, Turkey, Israel, Iran, Kuwait, Thailand, Malaysia, India, Korea, China, Australia, New Zealand). Almost and always were the females of *E. vermicularis* to be located in these ectopic sites. The ova appear to be considerably more resistant than the worm itself, and they remain well-preserved even when the latter is scarcely recognizable in the lesions or has completely disappeared. A good review of cases with such locations was already published in 1950 by Symmers [27] and then in 1991 by Sun et al. [35] and again in 2004 by Thomson [36].

Granulomas from *E. vermicularis* in the female genital tract usually represent a casual finding especially if asymptomatic. In symptomatic cases, they can cause bleeding, infertility and peritoneal adhesions; in still other cases, they can cause diffuse pelviperitoneal inflammation, tubal-ovarian abscesses responsible for hysterectomies, and/or ovariectomies, as in our case. In the differential diagnosis of these locations, granulomas of different origins can be taken into consideration: sarcoid, tuberculous, endometriotic, Crohn's disease, foreign body inflammatory reaction, due to the presence of different substances such as talc, silicon, and other surgical material, vegetable fragments from intestinal perforation, pilo-sebaceous material from broken ovarian dermoid cysts, malpighian squamae in which the keratin laminae derive from an ovarian and/or endometrial adenocarcinoma or from a squamous carcinoma of the cervix of the uterus. Finally, some granulomas correlated to the presence of *Schistosoma* spp. eggs in zones of endemic bilharziasis or in people coming from those zones can be observed.

Cases of perianal locations are less frequently reported; from the first case published by Froelich in 1897 [16] to the present case, the scientific literature has reported only 24 cases associated with *E. vermicularis*, mainly in males, frequently children and adolescents. A review of cases of enterobiasis in this location was reported in 1998 by Avolio et al. [37] Clinical manifestations are rarely absent. For the most part, the onset of the nodule is more or less painful, especially after defecation. It can be associated with a burning sensation or anal itching. The ulceration of the nodule is possible but has not been ascertained the nature of the event: causal or merely coincidental [25, 38].

Digital exploration of the rectum detects the presence of the nodule, more or less firm, painful at palpation, having a diameter of 1-2 cm. If it is not very painful, the nodule is tolerated by the patient, even for a number of months, before seeing a doctor (Vafai and Mohit, 1983) [18]; also see our case 2. Normally, eosinophilia is not reported. The diagnosis is based on the histological finding. In two cases, the diagnosis was cytological, with typical eggs having been found by means of fine needle aspiration [39, 40]. Among the other granulomatous lesions which may be confused with perianal lesions from *E. vermicularis* are those due to tubercular nodules, leiomyomatosis nodules, and granulomas from foreign body. If the patient comes from a zone endemic for bilharziasis, the differential diagnosis must include the granulomas caused by the eggs of *Schistosoma haematobium* or other species of trematodes of the same genus. Many cases located both in the female genital tract and in the ano-rectal zone could also be not diagnosed since sometimes notable symptoms are not present and other times due to the diagnostic error of the histopathologist.

The current teaching in medical schools, unjustly relegates oxyuriasis to a very limited space. But its impact on human health is not irrelevant, given the possible long duration of the infection, the possible obstinate resistance to the therapy and the possible ectopic locations which are difficult to diagnose clinically.

Nowadays, recent progress in the biology of pinworms based on techniques of extractive molecular biology can be used to overcome the difficulty of the clinical diagnosis and also to identify the provenance of the pinworm: from human patient or on the other hand from a captive chimpanzee. Moreover, molecular biology succeeded in clarifying the taxonomy of *Enterobius gregorii* by which Hugot 1983 supposed to be a new parasite for humans. Indeed, according to Nakano et al. 2006 [41], *gregorii*- and *vermicularis*-types are identical in the ITS2 region and intermingled in the clusters of the CO1 gene. Consequently, molecular evidence is that *E. gregorii* and *E. vermicularis* are two different names for the same pinworm.

## 4. Conclusions

By means of reporting the two ectopic cases described above, the authors wanted, among other things, to focus the attention of the medical world on this subject, shedding light on this unjustly neglected parasitosis, for the most part considered “out of fashion.”
